# RNAi Screen Reveals Host Cell Kinases Specifically Involved in *Listeria monocytogenes* Spread from Cell to Cell

**DOI:** 10.1371/journal.pone.0023399

**Published:** 2011-08-10

**Authors:** Ryan Chong, Raynal Squires, Rachel Swiss, Hervé Agaisse

**Affiliations:** Section of Microbial Pathogenesis, Boyer Center for Molecular Medicine, Yale University School of Medicine, New Haven, Connecticut, United States of America; Charité-University Medicine Berlin, Germany

## Abstract

Intracellular bacterial pathogens, such as *Listeria monocytogenes* and *Rickettsia conorii* display actin-based motility in the cytosol of infected cells and spread from cell to cell through the formation of membrane protrusions at the cell cortex. Whereas the mechanisms supporting cytosolic actin-based motility are fairly well understood, it is unclear whether specific host factors may be required for supporting the formation and resolution of membrane protrusions. To address this gap in knowledge, we have developed high-throughput fluorescence microscopy and computer-assisted image analysis procedures to quantify pathogen spread in human epithelial cells. We used the approach to screen a siRNA library covering the human kinome and identified 7 candidate kinases whose depletion led to severe spreading defects in cells infected with *L. monocytogenes*. We conducted systematic validation procedures with redundant silencing reagents and confirmed the involvement of the serine/threonine kinases, CSNK1A1 and CSNK2B. We conducted secondary assays showing that, in contrast with the situation observed in CSNK2B-depleted cells, *L. monocytogenes* formed wild-type cytosolic tails and displayed wild-type actin-based motility in the cytosol of CSNK1A1-depleted cells. Furthermore, we developed a protrusion formation assay and showed that the spreading defect observed in CSNK1A1-depleted cells correlated with the formation of protrusion that did not resolve into double-membrane vacuoles. Moreover, we developed sending and receiving cell-specific RNAi procedures and showed that CSNK1A was required in the sending cells, but was dispensable in the receiving cells, for protrusion resolution. Finally, we showed that the observed defects were specific to *Listeria monocytogenes,* as *Rickettsia conorii* displayed wild-type cell-to-cell spread in CSNK1A1- and CSNK2B-depleted cells. We conclude that, in addition to the specific host factors supporting cytosolic actin-based motility, such as CSNK2B, *Listeria monocytogenes* requires specific host factors, such as CSNK1A1 in order to form productive membrane protrusions and spread from cell to cell.

## Introduction

Various intracellular bacterial pathogens display actin-based motility within human cells, including *Listeria monocytogenes* and *Rickettsia conorii* [1], [2]. *L. monocytogenes* is a Gram-positive bacterium that invades epithelial cells of the intestinal mucosa and causes rare but potentially lethal food-borne infections. *R. conorii* is an obligate Gram-negative bacterium responsible for Mediterranean spotted fever (MSF), a disease transmitted to humans by the brown dog tick *Rhipicephalus sanguineus*. The ability to spread from cell to cell is an important determinant of virulence since infection in animal models with strains of *L. monocytogenes* and *R. rickettsii* impaired in actin-based motility leads to attenuated pathogenesis [3], [4], [5].

The bacterial and host factors supporting actin-based motility have been extensively investigated. Seminal genetic studies identified ActA as the bacterial factor required for *L. monocytogenes* actin tail formation [3], [4]. ActA displays structural similarities with WASP/WAVE family members, thereby mimicking their nucleation-promoting activity towards the ARP2/3 complex [6], [7], [8]. It was initially thought that, similar to *L. monocytogenes*, *Rickettsia spp.* actin-based motility relies on the expression of RickA, a bacterial factor that mimics the activity of WASP/WAVE family members [9], [10]. However, recent studies revealed that *R. rickettsii* actin-based motility is probably mediated by the formin-like bacterial factor, Sca2 [5].

The host factors involved in actin-based motility were primarily investigated by a biochemical approach using *L. monocytogenes* as a model system. Because the formation of actin tails could be observed in cells as well as in cell extracts, an *in vitro* system was used to identify the host factors required for actin tail formation [11], an approach that led to the identification of the ARP2/3 complex and its major role in actin nucleation [12]. Recently, a set of host factors required for *Rickettsia parkeri* actin tail formation was identified by a targeted approach using the *Drososphila* S2R+ cell line as a model system [13]. This study further revealed that, in order to display wild-type actin-based motility in COS-7 cells, *R. parkeri* requires the actin cytoskeleton factors Profilin and Fimbrin, two host factors that were seemingly dispensable for *Listeria monocytogenes* actin-based motility under these experimental conditions [13].

Whereas our understanding of cellular mechanisms supporting actin-based motility is now quite substantial, the host factors potentially supporting the formation of membrane protrusions are unknown. A common assumption postulates that the propelling force developed by bacteria displaying actin-based motility may be sufficient to deform the plasma membrane when moving bacteria reach the cell cortex. In support of this assumption, an *E. coli* strain engineered to express IcsA, the *Shigella* virulence factors supporting actin tail formation, was shown to display cytosolic actin-based motility and spread from cell to cell through membrane protrusion formation [14]. While this study suggests that the ability to develop actin-based motility may be the only requirement for the bacteria to protrude into the plasma membrane, the identity of the host factors potentially involved in the formation of productive protrusions and efficient spread from cell to cell is unknown.

Here, we present a kinome RNAi screen for host factors required for *L. monocytogenes* spread from cell to cell and report the identification and validation of the serine/threonine kinase CSNK1A1 and CSNK2B. We have previously shown that CSNK2B-mediated phosphorylation of ActA is required for cytosolic motility [15]. In contrast, we found that CSNK1A1 is not required for the development of cytosolic actin-based motility. We showed that CSNK1A1 depletion in the sending cells leads to a decrease in the resolution of membrane protrusions into double membrane vacuoles in the receiving cells, thus accounting for the observed cell-to-cell spreading defects. This study indicates that, in addition to their ability to display cytosolic actin-based motility, intracellular pathogens require specific host factors in order to form productive membrane protrusions and spread from cell to cell.

## Results

### Identification of *L. monocytogenes* spreading defects by high-throughput fluorescence microscopy and computer-assisted image analysis

**Figure 1:**
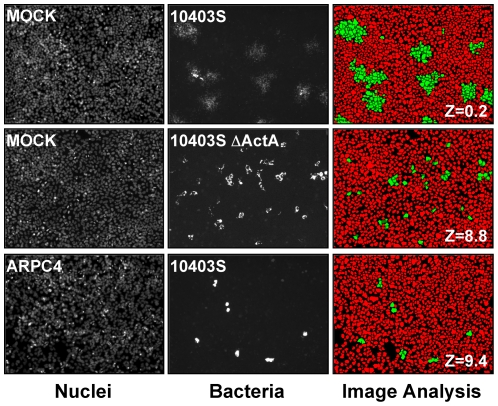
Fluorescence microscopy and image analysis of bacterial pathogen spread from cell to cell. Mock- and ARPC4-treated cells were infected with GFP-expressing wild type (*10403S*) or mutant (*10403SΔactA*) *L. monocytogenes* for 10 hrs. Left panel, Nuclei; middle panel, Bacteria and right panel, Image analysis. Computer-assisted image analysis was used to determine the number of nuclei corresponding to uninfected and infected cells (red and green objects, respectively).

The most widely used assay to study pathogen spread is the plaque assay. Macroscopic in nature, the plaque assay is not informative at the cellular level, nor is it amenable to high-throughput screening procedures. With the objective of identifying host factors involved in pathogen spread, we have developed a high-throughput screening strategy for quantification of *L. monocytogenes* spread in human epithelial cells using automated fluorescence microscopy and computer-assisted image analysis (Figure 1). In wild-type cells, GFP-expressing bacteria spread from cell to cell, forming characteristic infection foci (Figure 1, MOCK, *10403S*). As expected, the spreading-defective *ΔactA* mutant invaded cells as efficiently as the wild-type bacteria, but did not form the characteristic infection foci (Figure 1, MOCK, *10403SΔactA*). We used computer-assisted image analysis to determine the average intensity of the GFP signal (AI-GFP) in infected cells (Figure 1, Image analysis). For comparative analysis between samples, we used Z-scores to determine how many standard deviation units a given AI-GFP is above or below the mean (see Materials and Methods). Statistical analysis of hundreds of images demonstrated that more than 90% of wild-type cells infected with wild-type bacteria displayed AI-GFP dispersed within a 2 standard deviation interval, with corresponding Z-scores ranging from -2 to +2 (Figure 1, MOCK, *10403S*, Image analysis, Z = 0.2). We showed that the accumulation of non-spreading bacteria in primarily infected cells resulted in a dramatic increase of AI-GFP (Figure 1, MOCK, *10403SΔactA*, Image analysis, Z-score  = 8.8). Thus, the quantification of AI-GFP and the determination of the corresponding Z-scores provide a simple readout to identify spreading defects by computer-assisted image analysis.

### Identification of host factors required for *L. monocytogenes* spread by RNA interference

To test the feasibility of identifying host factors involved in cell-to-cell spread, we imaged *L. monocytogenes* infection in ARPC4-depleted cells. ARPC4 is a component of the ARP2/3 complex, a key cellular regulator of actin polymerization [16], [17]. We determined by real-time PCR analysis that siRNA treatment led to ∼90% depletion in ARPC4 transcript (not shown). As expected, in ARPC4-depleted cells, infection with wild-type bacteria displayed a spreading defect phenotype similar to the one observed in wild-type cells infected with the *ΔactA* mutant strain (Figure 1, ARPC4, *10403S*). Computer-assisted image analysis showed a dramatic increase of AI-GFP in ARPC4-depleted cells (Figure 1, Image analysis, ARPC4, Z-score  = 9.4). These results demonstrated the feasibility of using RNAi in combination with fluorescence microscopy and computer-assisted image analysis to identify host factors involved in *L. monocytogenes* spread from cell to cell.

### RNAi kinome screen and validation procedures

**Figure 2:**
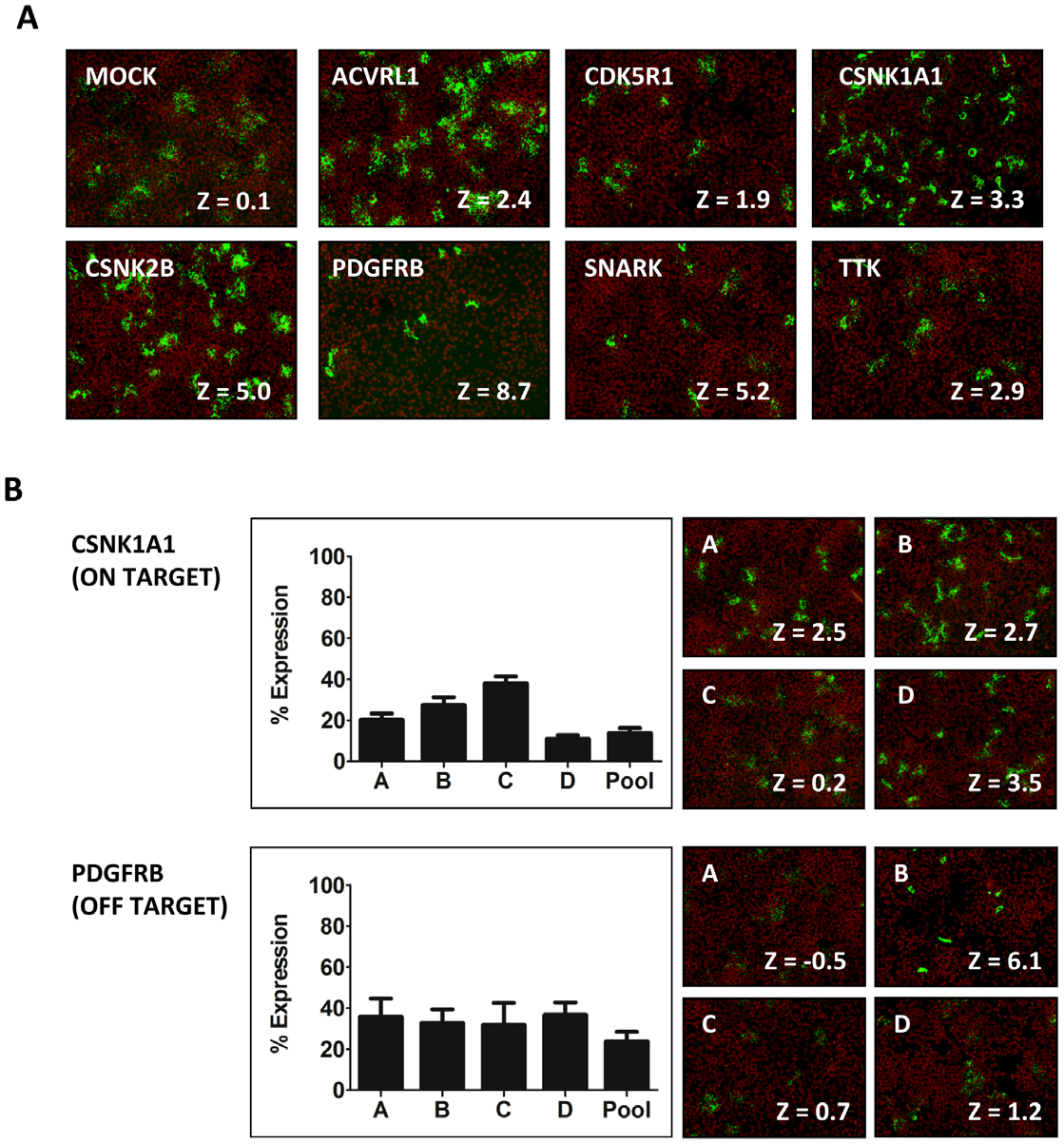
Primary screen and validation procedures. (A) Representative images for all candidate genes displaying a Z-score of ≥2. Merged images: red, Nuclei; green, Bacteria (B) Validation of individual siRNA duplexes and characterization of ON- and OFF-target candidates. Left panel: Real-time PCR analysis showing silencing efficiency compared to mock-treated cells (% Expression). Right panel: Representative images for the four individual siRNA duplexes tested and corresponding Z-scores. Merged image: red, Nuclei; green, Bacteria.

We next screened a siRNA library covering the human kinome (779 genes). The library was constituted of pools of four redundant silencing duplexes each targeting a given gene. Genes scoring two standard deviation units above AI-GFP were considered as potential hits (Z-score >2). We identified 7 candidate kinase genes that passed our selection criteria in three independent primary screens (ACVRL1, CDK5R1, CSNK1A1, CSNK2B, PDGFRB, SNARK and TTK). As part of our validation procedures, we re-tested the siRNA pools corresponding to the candidate genes and we confirmed the spreading defect phenotypes observed in the primary screen, with the exception of CDK5R1, which displayed a Z-score <2 (Figure 2A). A major drawback of the RNAi methodology is the unintended silencing of genes displaying limited sequence homology with the targeted gene, a phenomenon referred to as the off-target effect [18]. In order to establish a functional relationship between the silencing of the targeted genes and the observed spreading defects, we tested individually the four siRNA duplexes constituting the original pools and determined their silencing efficiency by real-time PCR and the strength of the conferred spreading defects by image analysis. For CSNK1A1 and CSNK2B, we observed a good correlation between Z-score values and silencing efficiencies (Figure 2B, CSNK1A1 and Table 1). These candidate genes are therefore likely to be specifically involved in the observed phenotypes (Fig. 2B, on-target). For the other candidate genes, however, our validation strategy revealed an extensive rate of false-positive hits (off-target). ACVRL1 was disregarded from further analysis because we could not detect any expression in HeLa 229 cells. For TTK, we confirmed that the pool of four siRNA duplexes conferred a spreading defect, as observed in the primary screen. However, none of the siRNA duplexes conferred a spreading defect when tested individually (Table 1). For ACVRL1, CDK5R1, PDGFRB and SNARK, only one siRNA duplex out of the 4 tested for each gene was found to confer the observed spreading phenotype, even though other duplexes displayed similar silencing efficiencies (Figure 2B, PGGFRB and Table 1). Since we could not establish an unambiguous relationship between the silencing of the targeted genes and the observed spreading defects, ACVRL1, CDK5R1, PDGFRB, SNARK and TTK were disregarded from further analysis (Fig. 2B, off-target and Table 1). Altogether our validation procedures suggest that 5 out of the 7 identified candidate genes were false-positive hits. In conclusion, our RNAi kinome screen revealed that CSNK1A1 and CSNK2B are specifically involved in *Listeria monocytogenes* spread from cell to cell.

**Table 1:** Hit validation and summary.

SNARK	RT-PCR	Z-Score	PDGFRB	RT-PCR	Z-Score
C	38.3±13.6	−0.15±0.4	C	31.6±11.0	0.68±0.2
A	69.5±17.1	−0.45±0.1	**B**	**32.6±6.8**	**6.06±2.3**
**B**	**72.0±27.7**	**4.36±0.9**	A	35.6±9.1	−0.49±0.4
D	72.3±14.1	−0.15±0.1	D	36.6±6.2	1.16±0.4
Pool	54.8±18.8	5.16±2.8	Pool	23.6±4.8	8.7±1.5

CSNKA1	RT-PCR	Z-Score	ACVRL1	RT-PCR	Z-Score
**D**	**10.8±1.9**	**3.46±0.5**	**B**	**NA**	**2.74±0.5**
**A**	**20.0±3.3**	**2.46±0.3**	C	NA	0.78±0.1
**B**	**27.2±4.0**	**2.65±0.5**	A	NA	−0.39±0.1
C	37.8±3.6	0.18±0.2	D	NA	0.33±0.2
Pool	13.6±2.6	3.34±0.9	Pool	NA	2.35±1.1

CSNK2B	RT-PCR	Z-Score	CDK5R1	RT-PCR	Z-Score
**C**	**16.2±4.1**	**5.2±0.2**	B	44.3±23.2	0.25±0.8
**A**	**16.4±2.2**	**6.0±1.1**	D	51.0±12.9	0.69±0.8
**D**	**20.5±2.9**	**2.8±0.4**	C	59.8±13.5	−0.26±0.1
**B**	**31.0±0.9**	**3.9±0.7**	**A**	**62.7±4.2**	**4.2±1.6**
Pool	18.2±1.7	5.0±0.2	Pool	51.8±14.4	1.94±0.8

TTK	RT-PCR	Z-Score	Validation Summary		
D	14.3±1.2	0.01±0.2	**ON-Target**	**OFF-Target**	
C	15.0±2.0	0.78±0.2	CSNK1A1	ACVRL1	PDGFRB
B	18.7±5.5	NA	CSNK2B	CDK5R1	TTK
A	40.3±2.3	0.12±0.2		SNARK	
Pool	10.3±0.6	2.93±1.3			

For each hit identified in the primary screens, four redundant siRNA duplexes were tested individually to determine the silencing efficiency by real-time PCR analysis (RT-PCR, % mRNA remaining) and the strength of the observed spreading phenotype (Z-score). In bold are the duplexes conferring a spreading defect (Z-Score >2). Only CSNK1A1 and CSNK2B targeting duplexes display a strict correlation between silencing efficiency and spreading defect phenotype and are therefore specifically involved in *L. monocytogenes* cell-to-cell spread. The bottom right corner displays the validation summary with 2 ON-target (true) and 5 OFF-target (false) hits.

### *L. monocytogenes* displays normal cytosolic actin-based motility in CSNK1A1-depleted cells

**Figure 3:**
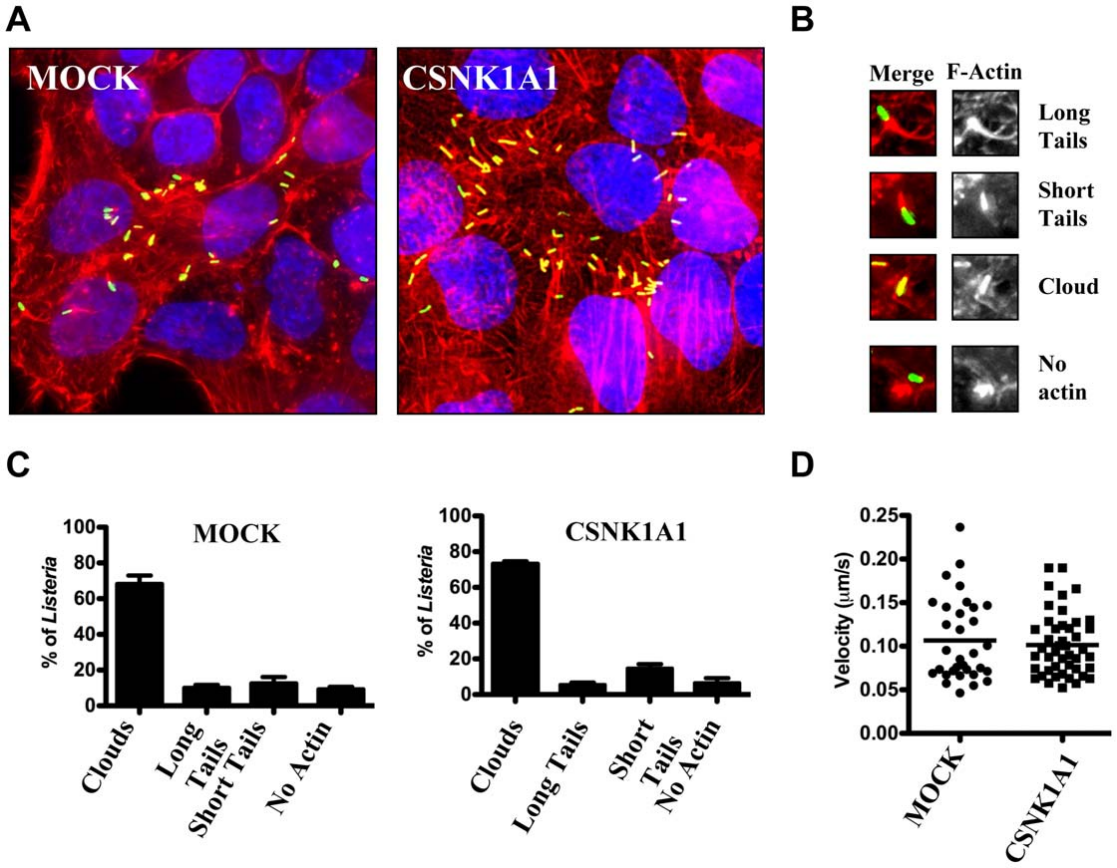
Characterization of actin-based motility in wild-type and CSNK1A1-depleted cells. (A) Representative images showing actin tails formed by *L. monocytogenes* in mock-treated and CSNK1A1-depleted cells. Merged image: green, Bacteria; red, F-actin; blue, DNA. (B) Representative images of the scored actin tail patterns. Left panel: Merged image: green, Bacteria; red, F-actin. Right panel: F-actin only. (C) Quantification of scored actin tail patterns as shown in (B) in mock-treated and CSNK1A1-depleted cells. Values represent the mean and standard deviation of 4 independent experiments. (D) Cytosolic velocity measurements in mock-treated and CSNK1A1-depleted cells.

To further investigate the role of CSNK1A1 in *L. monocytogenes* spread, we analyzed actin tail pattern formation in MOCK-treated and CSNK1A1-depleted cells (Fig. 3A). We scored bacteria that displayed long or short actin tails, or were surrounded by F-actin (clouds), or were not associated with F-actin (Fig. 3B). As opposed to the situation observed in CSNK2B-depleted cells, actin tail patterns formed in CSNK1A1-depleted cells were indistinguishable from the ones observed in mock-treated cells (Fig. 3C), suggesting that the bacteria displayed wild-type actin-based motility in CSNK1A1-depleted cells. We also conducted time-lapse video microscopy experiments and confirmed that the velocity of the bacteria in the cytosol of CSNK1A1-depleted cells was comparable to the velocity recorded in mock-treated cells (∼0.1 µm/s, Fig. 3D). We conclude that the spreading defect observed in CSNK1A1-depleted cells was not due to a detectable defect in cytosolic actin-based motility.

### *L. monocytogenes* forms non-productive membrane protrusions in CSNK1A1-depleted cells

**Figure 4:**
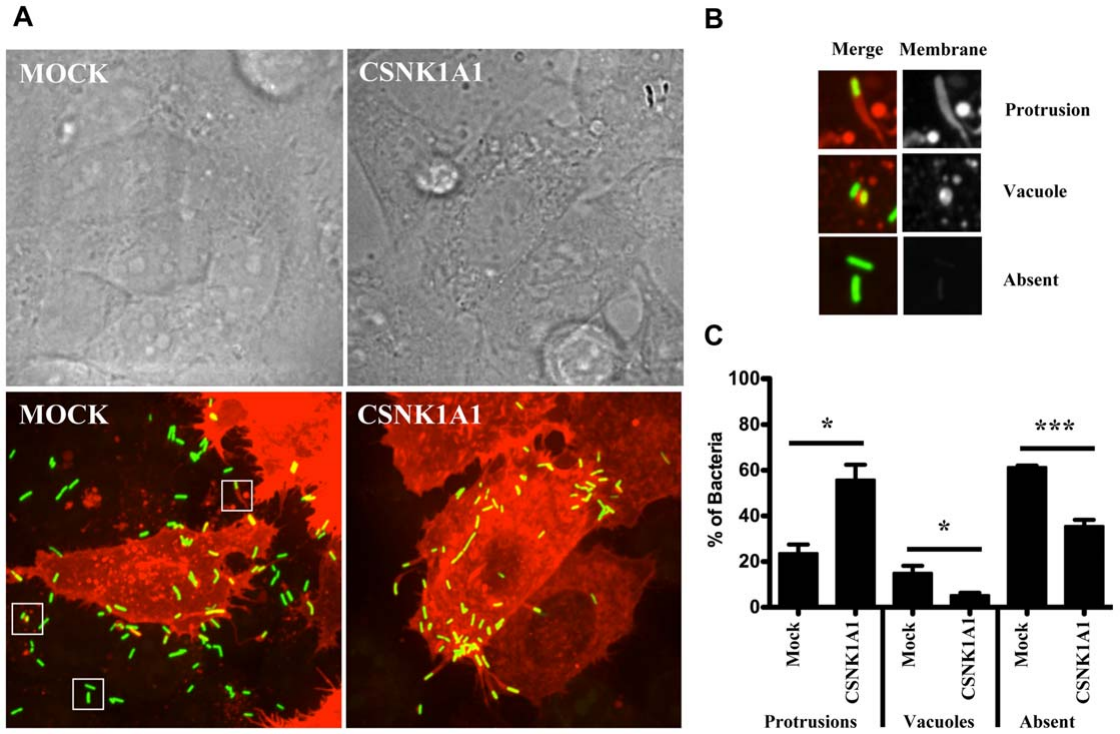
Characterization of membrane protrusion formation in mock-treated and CSNK1A1-depleted cells. (A) Representative images showing protrusion formation in mock-treated and CSNK1A1-depleted cells transiently transfected with membrane-targeted RFP. Top panel; bright field. Bottom panel; fluorescence (green, bacteria; red, plasma membrane). (B) Representative images of bacteria protrusions (top panel), double membrane vacuole (middle panel) and not associated with membrane marker (bottom panel). Left: merged image: green, bacteria; red, plasma membrane. Right: plasma membrane only. (C) Quantification of bacteria that crossed the cell boundary in mock-treated and CSNK1A1-depleted cells and found in protrusions, vacuoles or not associated with the membrane-RFP marker. Values represent the mean and standard deviation of 5 independent experiments.

Fluorescence microscopy analysis of actin tail pattern, as well as time-lapse video microscopy experiments, suggested that *L. monocytogenes* could move in the cytosol of infected cells and reached the plasma membrane after 4 hours of infection. However, as opposed to the situation observed in mock-treated cells (Fig. 4A, MOCK), *L. monocytogenes* did not spread to the neighboring cells in CSNK1A1-depleted cells (Fig. 4A, CSNK1A1). We used a plasma membrane-targeted version of RFP to test the hypothesis that, in CSNK1A1-depleted cells, the bacteria were not able to form productive membrane protrusions. This assay allowed us to identify, among the bacteria that crossed the cell boundary of an infected cell, the membrane marker-positive bacteria still present in membrane protrusions or double-membrane vacuoles and the membrane-marker-negative bacteria, that presumably reached the cytosol of the neighboring cells (Fig. 4B). We determined that in wild-type cells, ∼20% of the bacteria that crossed the cell boundary of an infected cell were found in protrusions (Fig. 4C, Mock, Protrusions). In stark contrast, ∼60% of the bacteria that crossed the cell boundary were located in protrusions in CSNK1A1-depleted cells (Fig. 4C, CSNK1A1, Protrusions). Interestingly, this accumulation of protrusions in CSNK1A1-depleted cells correlated with a significant decrease in the formation of double membrane vacuoles (Fig. 4C, CSNK1A1, Vacuoles), suggesting that the spreading phenotype observed in CSNK1A1-depleted cells was related to a defect in the resolution of protrusions into double membrane vacuoles. Accordingly, more than 60% and less than 30% of the bacteria that crossed the cell boundary were not associated with the membrane marker and therefore presumably reached the cytosol of the neighboring cells, in mock-treated CSNK1A1-depleted cells, respectively (Fig. 4C, Mock and CSNK1A1, Absent). These experiments indicate that, in CSNK1A1-depleted cells, *L. monocytogenes* forms abortive protrusions that do not resolve in double-membrane vacuoles.

### CSNK1A1 is required in sending cells, but not in receiving cells

**Figure 5:**
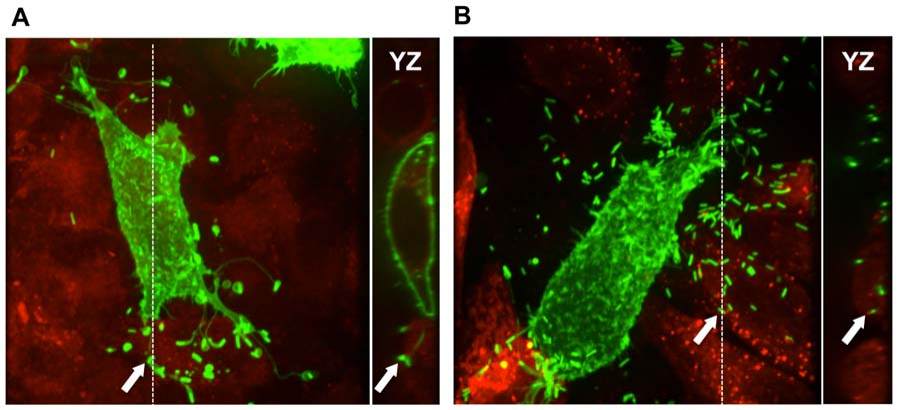
CSNK1A1 is required in sending cells, but not in receiving cells. (A) Representative image of CSNK1A1-depleted cells expressing membrane-GFP marker (green) infected with GFP-expressing *L. monocytogenes* and sending bacteria into mock-treated cells labeled with CMTPX (red). The doted line indicates the position of the YZ section shown on the right. Arrow indicates a protrusion in the CMTPX-labeled neighboring cell. (B) Representative image of mock-treated cells expressing membrane-GFP marker (green) infected with GFP-expressing *L. monocytogenes* and sending bacteria into CSNK1A1-depleted cells labeled with CMTPX (red). The doted line indicates the position of the YZ section shown on the right. Arrow indicates a free bacterium in the CMTPX-labeled neighboring cell.

We next determined the requirement for CSNK1A1 in the sending and receiving cells for efficient spread. To determine the requirement in the sending cells, we depleted CSNK1A1 in cells expressing a membrane-GFP marker and mixed them with mock-treated cells previously stained with a red cytosolic marker. CSNK1A1-depleted cells expressing membrane-GFP infected with *L. monocytogenes* displayed a typical spreading defect phenotype with formation of protrusions that failed to resolve in the receiving cells (Figure 5A). To determine the requirement in the receiving cells, we depleted CSNK1A1 in cells prior to labeling them with the red cytosolic dye and mixed them with mock-treated cells expressing the membrane-GFP marker. The protrusions sent by mock-treated cells into CSNK1A1-depleted cells were resolved as demonstrated by confocal microscopy (Figure 5B). These experiments indicate that CSNK1A1 is required in sending cells, but not in receiving cells for protrusion resolution.

### *Rickettsia conorii* displays normal cell-to-cell spread in CSNK1A1- or CSNK2B-depleted cells

**Figure 6:**
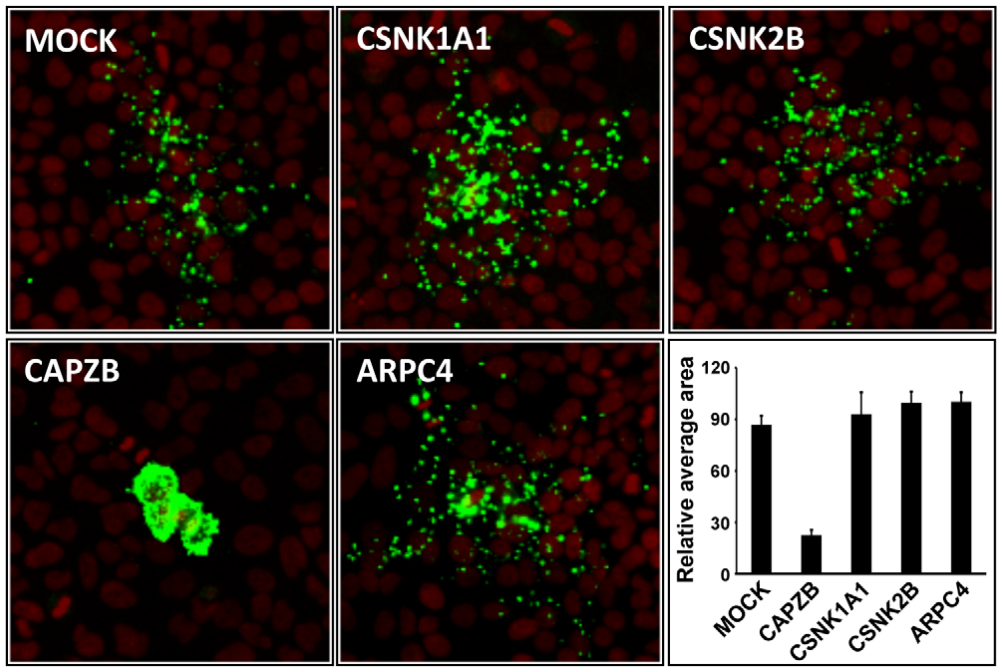
*R. conorii* spread from cell to cell does not rely on CSNK1A1, CSNK2B or ARPC4. Mock- or siRNA-treated cells were infected with *R. conorii* for 48 hrs and subjected to fluorescence microscopy. Red, Nuclei and green, Bacteria. The images show a representative example of the bacterial foci for each treatment. The graph shows quantification of the area covered by the bacterial foci for each treatment. Values represent the mean and standard deviation for 20 foci for each treatment. The area values were normalized with the highest value (ARPC4), which was given an arbitrary value of 100 (Relative average area).

In order to determine the specificity of the spreading defect phenotypes observed in CSNK1A1- and CSNK2B-depleted cells infected with *L. monocytogenes*, we tested the impact of CSNK1A1 or CSNK2B depletion on *Rickettsia conorii* spread from cell to cell. We first quantified *R. conorii* spread in mock-treated cells and in CAPZB-depleted cells. CAPZB is the beta subunit of Capping Z proteins, a cytoskeleton factor that was shown to be important for *R. conorii* actin-based motility [13]. *R. conorii* formed large infection foci in mock-treated cells (Fig. 6, MOCK). As expected, CAPZB depletion resulted in a striking decrease of the size of the infection foci (Fig. 6, CAPZB). We next analyzed *R. conorii* spread in CSNK1A1-, CSNK2B-depleted cells and determined that the infection foci formed in the corresponding cells were indistinguishable from the ones observed in wild-type cells (Fig. 6). Given the predicted role of RickA in *Rickettsia* spread and the predicted role of CSNK2B in the regulation of RickA affinity for the ARP2/3 complex, we also tested the potential role of the ARP2/3 complex in Rickettsia spread (Fig. 6, ARPC4). We found that the ARP2/3 complex is not required for Rickettsia spread in the cellular system under investigation. In conclusion, CSNK1A1, CSNK2B and the ARP2/3 complex are not required for *R. conorii* spread and are therefore specifically required for *L. monocytogenes* spread from cell to cell.

## Discussion

### RNAi screens and the rate of off-target effects in mammalian cells

We have developed a novel approach to quantify pathogen spread using high-throughput fluorescence microscopy and computer-assisted image analysis. We used the approach to screen a siRNA library covering the human kinome (779 genes) and identified 7 candidate genes involved in *L. monocytogenes* spread. In order to unambiguously establish a functional relationship between the silencing of the targeted gene and the observed phenotype, we required a strict correlation between silencing efficiency and the observed phenotype. To this end, we tested individually the 4 siRNA duplexes constituting the screened pools. For CSNK1A1 and CSNK2B, we found a total of 3 and 4 siRNA duplexes, respectively, conferring the observed phenotype and we could establish a perfect correlation with the silencing efficiency (Table 1). Therefore, our validation procedures revealed that CSNK1A1 and CSNK2B are specifically involved in *L. monocytogenes* spread. For ACVRL1, CDK5R1, PDGFRB, SNARK and TTK, however, we found only 1 duplex out of the 4 duplexes tested for each pool that conferred a spreading defect, and we could not establish any correlation between silencing efficiency and the observed phenotype. We conclude that the siRNA duplexes conferring a spreading defect phenotype for ACVRL1, CDK5R1, PDGFRB, SNARK and TTK probably display off-target activity. These results indicate that the rate of false-positive hits in our RNAi screen was extremely high (5/7 candidate genes, ∼70%), and that the validation procedure conducted using redundant silencing reagent was therefore essential in order to unambiguously demonstrate the involvement of the identified factors, including CSNK1A1 and CSNK2B.

### Host factors specifically involved in pathogen spread from cell to cell

Our comparative analyses with *R. conorii* revealed the specific requirement for CSNK1A1 and CSNK2B in *L. monocytogenes* spread from cell to cell. These two kinases, which were historically characterized by their ability to phosphorylate casein *in vitro* (hence their names), are in fact not related and display different substrate specificity. We have previously shown that CSNK2B mediates the phosphorylation of the C region of ActA, which regulates its affinity for the ARP2/3 complex [15]. On the basis of comparative analyses, we predicted that the activity of RickA, a *Rickettsia* protein shown to activate the ARP2/3 complex *in vitro*, would also be regulated by CSNK2B upon *Rickettsia* infection [15]. However, the data presented here indicate that *R. conorii* spreads normally in CSNK2B-depleted cells. Moreover, in agreement with a recent report showing that the ARP2/3 complex is not required for *R. parkeri* actin tail formation in *Drosophila* S2R+ cells [13], we showed that ARP2/3 is not required for *R. conorii* spread in human HeLa 229 cells. Finally, a recent report showed that a *R. rickettsii* mutant defective in the production of the putative outer membrane protein Sca2, which acts as a formin-like actin nucleator [19], displayed a spreading defect phenotype [5]. Collectively, these data suggest that the activity of the ARP2/3 complex and the nucleation-promoting factor RickA is not required for *R. conorii.* spread in HeLa 229 cells.

### CSNK1A1 is required for protrusion resolution into double membrane vacuoles

We previously showed that CSNK2B regulates actin-based motility in the cytosol of infected cells [15]. Here, we demonstrated that *L. monocytogenes* cytosolic motility was normal in CSNK1A1-depleted cells. Therefore, it is unlikely that CSNK1A1 regulate the activity of ActA in the cytosol of infected cells. However, we note that we cannot formally exclude the possibility that CSNK1A1-mediated phosphorylation regulates uncovered aspect(s) of ActA activity, not required for cytosolic actin-based motility, but necessary for spread from cell to cell. Similar to mock-treated cells, most bacteria were motile in the cytosol and could reach the plasma membrane in CSNK1A1-depleted cells. However, as opposed to the situation observed in mock-treated cells, the bacteria accumulated at cell boundaries, suggesting that they were not able to effectively spread from cell to cell. To further investigate the nature of the observed spreading defect, we developed a protrusion formation assay using a membrane-targeted version of RFP to visualize *L. monocytogenes*-containing plasma membrane protrusions. In addition, we developed a secondary assay to determine whether CSNK1A1 was required in the sending and/or the receiving cells. Altogether, our results indicate that CSNK1A1 is specifically required in the sending cells for the resolution of membrane protrusions into double-membrane vacuoles in the receiving cells.

### Mechanisms of protrusion resolution into double membrane vacuoles

The resolution of protrusions into vacuoles is a poorly understood process that requires disruption of the cytoskeleton network constituting the protrusions and subsequent sealing of the plasma membranes surrounding the protrusions. We speculate that these drastic remodeling events may rely on bacterial as well as cellular factors. *L. monocytogenes* produces three factors known to disrupt eukaryotic membrane integrity, including the pore-forming Listeriolysin O (LLO) [20], [21], the PI-specific phospholipase A (PlcA) [22], [23] and the broad-range phospholipase PlcB [3], [24]. PlcB also displays membrane fusion activity that could be involved in the resolution process [25]. It is also likely that the forces generated by *L. monocytogenes*-mediated actin polymerization in protrusions together with the disruptive activities of the bacterial factors aforementioned contribute to membrane tension and protrusion resolution. Whether CSNK1A1 may be involved in the regulation of these bacterial activities in protrusions remains to be investigated. The cellular factors potentially involved in protrusion resolution are unknown, but the nature of cell-cell contact is likely to be important. Interestingly, the *L. monocytogenes* factors InlC has been recently shown to be required for disrupting Tuba-mediated apical junctions in polarized cells (Caco-2), thereby releasing cortical tension at the plasma membrane and facilitating cell-to-cell spread [26]. It is therefore tempting to speculate that CSNK1A1 may be required for this process. However, the release of the apical tension for efficient spread is only important in polarized cells and there is no detectable spreading defect in non-polarized cells, such as the HeLa 229 cell line used in our study, when infected with the *InlC* mutant (Keith Ireton, personal communication). Since CSNK1A1 is a component of the complex controlling the levels of the adherens junction component β-catenin [27], we also tested the hypothesis that the spreading defect observed in CSNK1A1-depleted cells may be related to β-catenin stabilization. To this end, we conducted double knock-down experiments in order to decrease the expression of β-catenin in CSNK1A1-depleted cells. Silencing β-catenin expression had no impact on *L. monocytogenes* spread and silencing both β-catenin and CSNK1A1 expression did not rescue the spreading defects observed in CSNK1A1-depleted cells, suggesting that β-catenin stabilization is not the cause of the observed phenotype. CSNK1A1 displays numerous cellular substrates [27] and further investigation will be required to determine the potential role(s) of CSNK1A1 in *L. monocytogenes* protrusion resolution.

## Materials and Methods

### Bacterial and eukaryotic cell growth conditions

*Listeria monocytogenes* strain 10403S [28] was grown overnight in BHI (Difco) at 30°C without agitation prior to infection. *Rickettsia conorii* Brumpt, Strain 7 (ATCC # VR-613) was amplified in Vero cells (ATCC #CCL-81) as previously described [29]. HeLa 229 cells (ATCC #CCL-2.1) were grown in DMEM (Invitrogen) supplemented with 10% FBS (Gibco) at 37°C in a 5% CO_2_ incubator.

### RNAi screen

The Dharmacon library (ThermoFisher) covering the human kinome (779 genes) was aliquoted in black clear-bottom 384-well plates (Corning, 3712) by dispensing 10 µl of the corresponding siRNA pools (200nM). 20 µl of Dharmafect 1 transfection reagent (ThermoFisher) was diluted in 4 ml of DMEM serum free media (Invitrogen) and 10 µl of the mixture were dispensed to each well and incubated at room temperature for 20 min. Hela 229 cells were trypsinized, lifted and re-suspended in DMEM high glucose (Invitrogen) supplemented with 20% FBS (Gibco) at 100,000 cells/ml and 20 µl of the suspension were delivered to each well. The plates were then incubated at 37°C, in 5% CO2 for 3 days. After 3 days of RNAi treatment, the cells were infected (MOI = 20) with *L. monocytogenes* strain (10403S) expressing GFP under the control of a IPTG-inducible promoter. The plates were centrifuged at 1,000 rpm for 5 minutes and internalization of the bacteria was allowed to proceed for 1 hour at 37°C before gentamicin (50 µM final) and IPTG (10 mM final) were added in order to kill the remaining extra-cellular bacteria and visualize internalized bacteria. The cells were incubated at 37°C for 9 hours and then fixed in PBS 4% formaldehyde at 4°C overnight. The cells were stained with Hoechst 33348 (Molecular Probes, Inc.), washed in PBS and kept at 4°C until imaging.

### High-throughput imaging and computer-assisted image analysis

384-well plates were imaged using a TE 2000 microscope (Nikon) equipped with a Orca ER Digital CCD Camera (Hamamatsu), motorized stage (Prior), motorized filter wheels (Sutter Instrument, Inc.) and a 10X objective (Nikon) mounted on a piezo focus drive system (Physik Instrumente). Image acquisition and analysis were conducted using the MetaMorph 7.1 software (Molecular Devices, Inc.). The number of nuclei per field of view was determined by segmentation analysis of images captured in the DAPI channel. The number of infected cells was quantified by analysis of the GFP signal in the area encompassing the identified nuclei using the Cell Scoring module of the MetaMorph imaging software. The spreading process was scored by the determination of the average intensity of the GFP signal (AI) in infected cells. We determined that the standard deviation (SD) for wild-type cells infected with wild-type bacteria never exceeded +/− 20% of the AI value. Normalization and comparison among replicate experiments was conducted by attributing Z scores: Z =  (AI (well)- AI (plate))/SD), where AI(well) is the AI value for a given well and AI(plate) is the average of AI values across the entire 384-well plate. Candidate genes displaying a Z score >2, corresponding to two standard deviation units above AI(plate), in three independent primary screens, were considered as potential hits. As a reference example, Z = 9.4 for ARPC4 siRNA (Figure 1).

### Validation procedure

HeLa 229 cells were transfected by reverse transfection with Dharmafect 1 and individual siRNA (A, B, C and D, 50 nM final) or a pool of the four silencing reagents (12.5 nM each, 50nM total) and incubated for 72hrs on coverslips in a 24-well plate format. For real-time PCR analysis, total RNA and first-strand cDNA synthesis was performed using the TaqMan Gene Expression Cells-to-Ct Kit (Applied Biosystems), as recommended by the manufacturer, with the addition of DNaseI for the removal of unwanted genomic DNA. mRNA levels were determined by quantitative real-time PCR using the Universal ProbeLibrary (Roche, Biochemicals, Indianapolis, IN) and LightCycler 480 Probes Master (Roche, Biochemicals, Indianapolis, IN). Thermal cycling was carried out using a Light Cycler 480 instrument (Roche Diagnostics) under the following conditions: 95°C for 5 min and 45 cycles at 95°C for 10 s and 60°C for 25 s.

### Quantification of *L. monocytogenes* actin tail formation

HeLa 229 cells infected with *L. monocytogenes* were fixed in PBS 4% paraformaldehyde overnight. The cells were then permeabilized and stained in PBS/0.1% TritonX containing Alexa-fluor 568 conjugated phalloidin (1∶500) (Invitrogen), and Hoechst (1∶500) (Invitrogen) for one hour. The samples were then mounted onto glass slides using FluoroGuard Antifade Reagent (Bio-Rad) and imaged immediately using a TE 2000 microscope (Nikon) equipped with a Orca ER Digital CCD Camera (Hamamatsu), motorized stage (Prior), motorized filter wheels (Sutter Instrument, Inc.) and a 60X objective (Nikon) mounted on a piezo focus drive system (Physik Instrumente). Image acquisition and analysis was conducted using the MetaMorph 7.1 software (Molecular Devices, Inc.).

### Time-lapse video microscopy

HeLa 229 cells were seeded on 35-mm imaging dishes (MatTek, Ashland, MA). Mock- and siRNA-treated cells were grown on the same imaging dishes using a Culture-Insert (Ibidi, Germany). Cells were then transfected with pDsRed-Monomer-Mem DNA construct (Clontech) 18hrs prior to infection with GFP-expressing *L. monocytogenes*. At 4hrs post infection, images were captured every 8 sec on a Nikon TE2000E spinning disc confocal microscope. Single bacterium tracking was performed using the tracking module of the Volocity software (Improvision, Lexington, MA). Bacteria tracked to calculate average velocity were required to be motile for at least 30 sec and show a minimum velocity of 0.03 µm/sec.

### Quantification of *L. monocytogenes* membrane protrusion and vacuole formation

Mock- and siRNA-treated HeLa 229 cells were seeded on Day 1 on coverslips as described previously [15]. Cells were transfected with pDsRed-Monomer-Mem DNA construct (Clontech) on Day 3. Media was replaced on Day 4 and the cells were subsequently infected with GFP expressing-*L. monocytogenes* for 7hrs, fixed in PBS 4% para-formaldehyde. Coverslips were mounted onto glass slides using FluoroGuard Antifade Reagent (Bio-Rad) and imaged on a Nikon TE2000 spinning disc confocal microscope with a 60x oil objective. Images were processed using the Volocity software package (Improvision/PerkinElmer).

### Respective role of sending and receiving cells

Mock- and siRNA-treated HeLa 229 cells were cultured on glass coverslips in 24-well plates for 72 h as previously described [15]. 24 hours after transfection, mock- or siRNA-treated cells were either transfected with 200 ng pAcGFP1-MemHyg DNA construct (Clontech) or incubated with 2.5 µM CellTracker Red CMTPX (Molecular Probes, Invitrogen) for 15 min, washed with PBS and fresh DMEM 10% FBS was added. After 6hrs of incubation, the cells were washed with PBS again, supplemented with fresh DMEM 10% FBS and incubated overnight. The next day, both cell populations were lifted, mixed, plated onto coverslips and cultured overnight at 37°C. The mixed population of cells was infected the next day with GFP-expressing *L. monocytogenes* for 7 hours. Coverslips were imaged by spinning disc confocal microscopy as described for the quantification of protrusion and vacuole formation.

### *Rickettsia conorii* infection and quantification of spread from cell to cell

Mock- and siRNA-treated HeLa 229 cells were cultured on glass coverslips in 24-well plates for 72 h as described previously [15]. At the time of infection, cells were refreshed with DMEM 10% FBS containing *R. conorii* and incubated for 48 h at 37°C. The cells were fixed in PBS 4% paraformaldehyde. For immunostaining, cells were permeabilized and stained by incubating in PBS/0.1% Triton X-100/1% BSA containing a primary rabbit polyclonal antibody against whole *R. rickettsii* (a gift from R. Heinzen, Rocky Mountain Laboratories, USA) for 1 h at room temperature, followed by incubation with goat anti-rabbit Alexa-488 secondary antibody (Invitrogen) and Hoechst (Invitrogen) for 30 min. Coverslips were mounted on glass slides using FluoroGuard Antifade Reagent (Bio-Rad). The area covered by a given focus of infection was determined by using the Image Morphometry Analysis (IMA) module of the MetaMorph 7.1 software (Molecular Devices, Inc.).
